# GPO-VAE: modeling explainable gene perturbation responses utilizing GRN-aligned parameter optimization

**DOI:** 10.1093/bioinformatics/btaf256

**Published:** 2025-07-15

**Authors:** Seungheun Baek, Soyon Park, Yan Ting Chok, Mogan Gim, Jaewoo Kang

**Affiliations:** Department of Computer Science and Engineering, Korea University, Seoul, 02841, South Korea; LG AI Research, Seoul, 07789, South Korea; Department of Computer Science and Engineering, Korea University, Seoul, 02841, South Korea; Department of Computer Science and Engineering, Korea University, Seoul, 02841, South Korea; Department of Biomedical Engineering, Hankuk University of Foreign Studies, Yongin, 17035, South Korea; Department of Computer Science and Engineering, Korea University, Seoul, 02841, South Korea; AIGEN Sciences, Seoul, 04778, South Korea

## Abstract

**Motivation:**

Predicting cellular responses to genetic perturbations is essential for understanding biological systems and developing targeted therapeutic strategies. While variational autoencoders (VAEs) have shown promise in modeling perturbation responses, their limited explainability poses a significant challenge, as the learned features often lack clear biological meaning. Nevertheless, model explainability is one of the most important aspects in the realm of biological AI. One of the most effective ways to achieve explainability is incorporating the concept of gene regulatory networks (GRNs) in designing deep learning models such as VAEs. GRNs elicit the underlying causal relationships between genes and are capable of explaining the transcriptional responses caused by genetic perturbation treatments.

**Results:**

We propose GPO-VAE, an explainable **VAE** enhanced by **G**RN-aligned **P**arameter **O**ptimization that explicitly models gene regulatory networks in the latent space. Our key approach is to optimize the learnable parameters related to latent perturbation effects toward GRN-aligned explainability. Experimental results on perturbation prediction show our model achieves state-of-the-art performance in predicting transcriptional responses across multiple benchmark datasets. Furthermore, additional results on evaluating the GRN inference task reveal our model’s ability to generate meaningful GRNs compared to other methods. According to qualitative analysis, GPO-VAE possesses the ability to construct biologically explainable GRNs that align with experimentally validated regulatory pathways.

**Availability and implementation:**

GPO-VAE is available at https://github.com/dmis-lab/GPO-VAE.

## 1 Introduction

Predicting cellular responses is crucial for understanding biological systems and complex cellular behavior, enabling the rational manipulation of cells to develop targeted therapeutics and improve treatments for various diseases. To address the task, many computational methods have been introduced in the realm of perturbation modeling (Gavriilidis *et al.* 2024). Generative modeling approaches have emerged as a key research focus in computational gene perturbation response prediction tasks (Sohn *et al.* 2015, Lopez *et al.* 2023, Bereket and Karaletsos 2024). In particular, variational autoencoders (VAEs) have become popular deep-learning model choices as they can learn latent features underlying perturbation responses from observed perturbation data during their training process.

However, the complex nature of perturbation experiments, including cell-specific contexts and technical variations, presents several challenges for improving the VAE’s generalizability. Disentangled VAEs, with their ability to learn disentangled representations despite these complexities, have become promising tools in perturbation modeling (Bereket and Karaletsos 2024). The main intuition is to enable VAEs to effectively disentangle and learn distinct latent subspaces such as those corresponding to perturbation effects and cell basal states. Notable models that pioneered this design approach are SVAE+ (Lopez *et al.* 2023) and SAMS-VAE (Bereket and Karaletsos 2024), both of which demonstrated success in improving perturbation response predictions through disentanglement of latent subspaces. Recently, *Baek et al.* tackled the issue of technical artifacts that affect experimental perturbation data quality and introduced CRADLE-VAE, a model that additionally learns a latent subspace related to artifacts utilizing counterfactual reasoning. This approach not only enhanced the prediction accuracy and robustness of the model but also improved its generative quality.

The main key design element in these VAEs is the Sparse Mechanism Shift hypothesis (Schölkopf *et al.* 2021). This hypothesis assumes that when a gene perturbation treatment is applied to a cell, only a few proportion of biological causal mechanisms contribute to the overall transcriptional gene expression changes. Encouraging sparsity on the latent features corresponding to gene perturbation effects has demonstrated robustness in predicting perturbation responses.

However, explainability remains a crucial element in biological and healthcare AI, particularly from medical, legal, and ethical perspectives (Amann *et al.* 2020). In fact, VAEs have limited explainability due to its learned latent representations lacking clear and direct associations with specific biological processes (Charte *et al.* 2020).

One promising approach to address these explainability issues is to incorporate the concepts of gene regulatory networks (GRNs) in the core design of VAEs. GRNs provide a mechanistic framework for understanding and predicting how cells respond to genetic perturbations. By aligning the latent subspace related to gene-specific perturbation effects with gene-gene causal relationships, it is possible to retain the benefits of sparsity while enhancing model explainability. Furthermore, guiding the learning trajectories of VAEs toward formation of GRNs derived from perturbation dataset may provide valuable insights related to cell-specific biological pathways.

In this work, we introduce GPO-VAE a VAE that exploits the concepts of gene regulatory networks for enhanced explainability. While retaining the Sparse Mechanism Shift hypothesis as a fundamental principle, we redesign the latent subspace responsible for modeling gene perturbation effects so that it aligns with GRN topologies. Specifically, we implemented GRN-Aligned Parameter Optimization, that guides the stochastic learning of prior parameters for sampling sparsity-inducing masks toward biologically explainable GRNs. While maintaining competitive performance in predicting transcriptional responses to perturbations, we demonstrate that the GRN constructed by GPO-VAE not only explains the perturbation data but also effectively captures important regulatory pathways that align with experimentally validated biological pathways.

## 2 Materials and methods

### 2.1 Datasets

We prepared three Perturb-Seq datasets [**Replogle K562** (Replogle *et al.* 2022), **Replogle RPE1** (Replogle *et al.* 2022), and **Adamson** (Adamson *et al.* 2016)]. All three datasets contain both observational (control) and interventional (post-perturbation) single-gene perturbation data instances. The gene perturbation dataset containing *N* data instances is a tuple consisting of D=(X,P,A), where $X\in R^{N\times |G|}$, $P\in {\left\{0,1\right\}}^{N\times |G^{{}^{\circ}}\cup G^{+}|}$, $A\in {\left\{0,1\right\}}^{N\times 1}$ correspond to the matrices of gene expression profiles, one-hot encoded gene perturbation treatments and quality control (QC) labels respectively.

Table 1 shows the detailed gene counts of each dataset used in this study. Given the total population of genes G used in each dataset, where each represents a gene expression value, we denote $G^{{}^{\circ}}$ as the perturbation gene subset and $G^{+}$ as the extended gene subset. The former contains perturbed genes for which interventional data is available, while the latter consists of additional extended genes with differential expression cutoff based on log FC and adjusted p-value that were not experimentally perturbed. We expanded the gene set to increase the coverage of genetic perturbations in our model.

**Table 1.** Details of each gene perturbation dataset used in this study.^a^

Dataset	No. of data instances D	No. of all genes G	No. of perturbed genes $G^{{}^{\circ}}$	No. of extended genes $G^{+}$
Replogle K562	129 478	8563	622	924
Replogle RPE1	91 891	8749	383	272
Adamson	46 236	17 035	68	347

a Note that $G^{{}^{\circ}},G^{+}\subset G$ while $G^{{}^{\circ}}\cap G^{+}=\emptyset$.

We adopted the preprocessing strategies from CausalBench, and further excluded samples annotated as *weak perturbation* as they are likely to represent unsuccessful knockdowns, which could introduce noise and compromise the robust evaluation of GRNs (Replogle *et al.* 2020, Chevalley *et al.* 2025). As our model is an extension of CRADLE-VAE, we adopted their method for annotating the gene expression profiles with quality control (QC) pass labels using six criteria, originally stated in the filtering guidelines by Scanpy and 10X Genomics (Baek *et al.* 2025). Details related to annotation of weak perturbation and QC labels are available in Supplementary Material S1.

### 2.2 Model architecture

GPO-VAE is a generative model that integrates the capabilities of gene perturbation response prediction and GRN inference-based explainability. It inherits the fundamental design principles from CRADLE-VAE including latent disentanglement, sparsity assumption of perturbation effects and counterfactual reasoning. Similar to conventional VAEs, GPO-VAE consists of an Encoder-Decoder architecture with its encoder module composed of three submodules—Latent Perturbation Encoder, Latent Artifact Encoder and Latent Basal State Encoder. These submodules construct latent embeddings for gene perturbation treatment, artifacts effects, and cell basal states, respectively. We modified the structure of latent features which determines the sparsity of the treatment effect so that the features represent GRNs governing the causal relations between genes. During the generative process, GPO-VAE takes a gene perturbation treatment vector as input and iteratively generates predicted cellular responses to compute average treatment effects.

**Figure 1.**
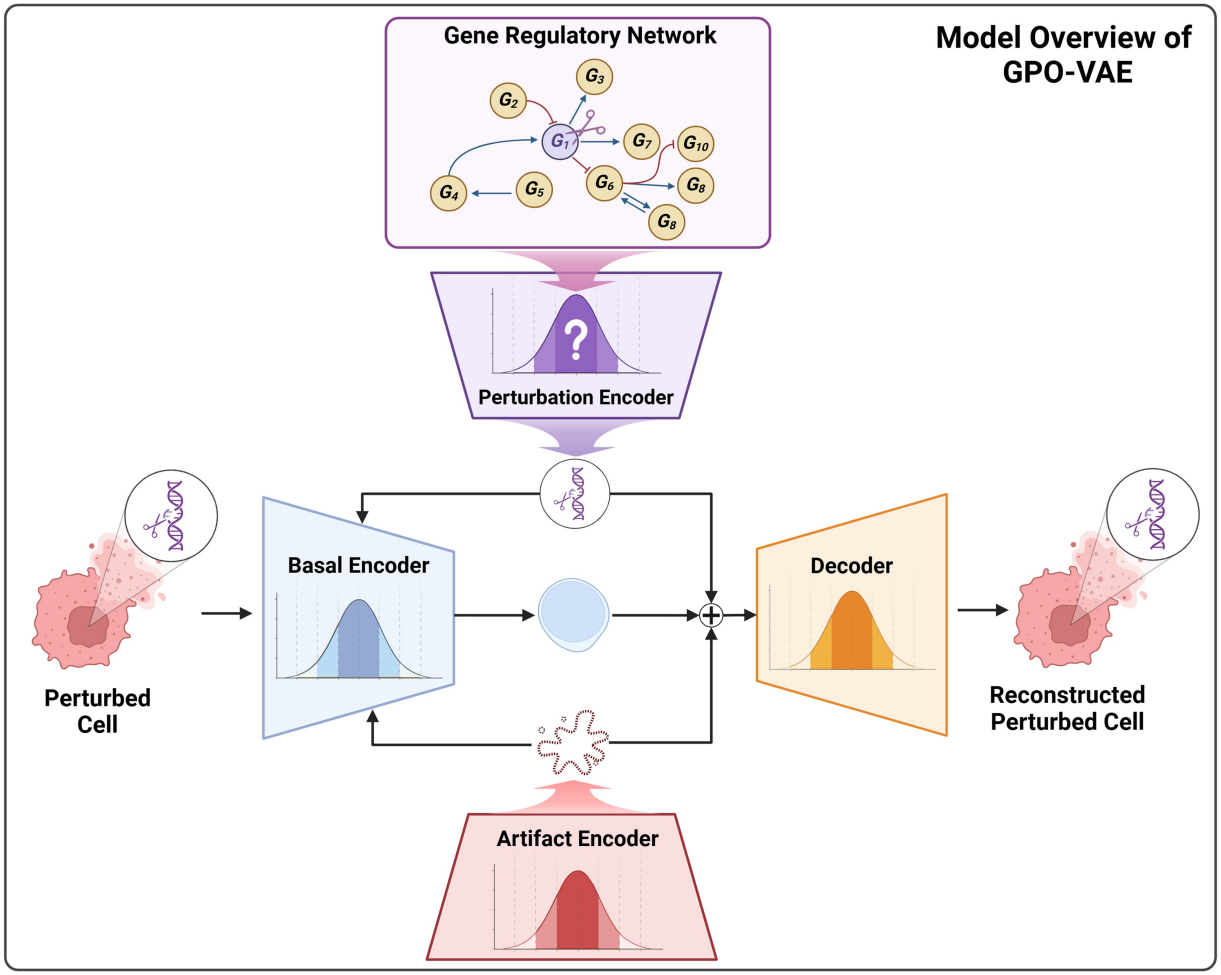
Model overview of GPO-VAE. The model consists of three encoder modules: latent perturbation encoder, latent artifact encoder, and latent basal state encoder, and a decoder. The perturbation encoder utilizes a gene regulatory network. Created with BioRender.com.

Figure 1 provides an illustrated overview of GPO-VAE’s model architecture. For clarity, we first describe the training process of each submodule, followed by the generative process.

#### 2.2.1 Encoder

The Latent Perturbation Encoder aims to learn the distribution of perturbation effects, independent of biological variation and artifact effects in a cell. During training, a global sparse latent offset matrix, $M\in {\left\{0,1\right\}}^{\left|G^{{}^{\circ}}\cup G^{+}|\times |G^{{}^{\circ}}\cup G^{+}\right|}$, is first sampled from the respective Bernoulli distribution with trainable parameters $\hat{W}\in R^{\left|G^{{}^{\circ}}\cup G^{+}|\times |G^{{}^{\circ}}\cup G^{+}\right|}$. Then, the sampled latent offsets are fed to parameterize the global latent gene-wise perturbation effect matrix, $E\in R^{\left|G^{{}^{\circ}}\cup G^{+}|\times |G^{{}^{\circ}}\cup G^{+}\right|}$, forming a Normal distribution, from which each latent perturbation effect is sampled. The latent gene perturbation effect embeddings, $Z_{p}$, for each data instance are constructed by combining E with M and indexing them by the gene perturbation treatment *P*.

The Latent Perturbation Encoder that takes the gene perturbation treatments of *N* data instances as input $P\in {\left\{0,1\right\}}^{N\times |G^{{}^{\circ}}\cup G^{+}|}$ is mathematically expressed as follows:


(1) $$M∼B(\hat{W})$$

(2) $$E∼N({\hat{f}}_{\text{enc},p}(M))$$

(3) $$Z_{p}=P(E⊙M)$$

where B(·) and N(·) represents Bernoulli distribution and Normal distribution, respectively. ${\hat{f}}_{\text{enc},p}$ denotes a trainable neural network which parameterizes the distribution from which M is sampled.

Leveraging counterfactual reasoning, we train the Latent Artifact Encoder to target quality issues through reinforcing the disentanglement of latent variables associated with the presence of technical artifacts. The encoder takes one-hot QC labels, $A\in {\left\{0,1\right\}}^{N\times 1}$, as input and models the distribution of technical artifacts independently of basal cell features and perturbations as a Normal distribution:


(4) $$Z_{a}=Au,\;\text{where}\;u∼N(\hat{\mu },\hat{\sigma }),$$

where u represents the global latent artifact embedding sampled from a parameterized Normal distribution, and $Z_{a}\in R^{N\times d}$ denotes the latent artifact embedding matrix for *N* data instances. As this is not the primary focus of our work, further details, including the counterfactual reasoning-based artifact disentanglement, are in the Supplementary Material S2.1.

The Latent Basal State Encoder builds latent basal state embeddings $Z_{b}\in R^{N\times d}$ based on integration of input gene expression profiles X, latent perturbation effect embeddings $Z_{p}$ and latent artifact embeddings $Z_{a}$.


(5) $$Z_{b}∼N({\hat{f}}_{\text{enc},b}(X,Z_{p},Z_{a})),$$

where ${\hat{f}}_{\text{enc},b}$ denotes a trainable neural network which parameterizes the distribution from which $Z_{b}$ is sampled.

#### 2.2.2 Decoder

In decoding, we integrate the distinct latent embedding matrices $Z_{b}$, $Z_{p}$, and $Z_{a}$, to reconstruct the input gene expression profile $\tilde{X}$. The reconstruction output $\tilde{X}\in R^{N\times |G|}$ is sampled from a neural network-equipped Gamma-Poisson distribution. The decoding process is expressed as follows:


(6) $$\Lambda ∼\Gamma ({\hat{f}}_{\text{dec}}(Z_{b},Z_{p},Z_{a})L,\Theta )$$

(7) $$\tilde{X}∼P(\Lambda )$$

where Γ(.) is the Gamma Distribution sampler, ${\hat{f}}_{\text{dec}}$ denotes the decoder-specialized neural network, P(.) is the Poisson Distribution sampler which is parameterized by $\Lambda \in R^{N\times |G|}$, $L\in R^{N\times 1}$ and $\Theta \in R^{N\times |G|}$ represent the total number of read counts in each cell and trainable parameters, respectively.

#### 2.2.3 Variational inference

Given the intractability of the data marginal likelihood p(X|P,A), we introduce the correlated variational distribution q(Z|X,P,A) as an approximation to the posterior distribution of the latent variables. This distribution is defined as:


(8) $$\begin{array}{cc} & q(Z_{b},M,E,U|X,P,A)=\left(\prod_{t=1}^{\left|G^{{}^{\circ}}\cup G^{+}\right|}q(e_{t}|m_{t};\phi )q(m_{t};\phi )\right)\\ & \;\times q(u;\phi )\left(\prod_{n=1}^{N}q(z_{b,n}|x_{n},p_{n},a_{n},M,E,U;\phi )\right) \end{array}$$

To approximate the posterior distribution log p(X|P,A), we apply stochastic variational inference. The learnable parameters (θ,ϕ) of GPO-VAE are optimized by maximizing the evidence lower bound (ELBO), which is given by the following expression:


(9) $$\begin{array}{cc} & J_{\mathrm{rec}}(\theta ,\phi )=E_{Z_{b},M,E,U∼q(\cdot |X,P,A;\phi )}\\ & \;\left[\text{log}\;\frac{p(X,Z_{b},M,E,U|P,A;\theta )}{q(Z_{b},M,E,U|X,P,A;\phi )}\right] \end{array}$$

#### 2.2.4 Generative process

For generation process after training, GPO-VAE samples latent basal state $Z_{b}$ from a Normal distribution N(0,I):


(10) $$Z_{b}∼N(0,I)$$

It is then concatenated with the latent artifact embedding $Z_{a}$, and latent gene perturbation effect embeddings $Z_{p}$ sampled from their respective parameterized distributions. The sampling process of $Z_{p}$ is the same as (1, 2), and (3). Notably, unlike the training process, A is a zero matrix, representing the absence of artifacts to generate artifact-free samples:


(11) $$Z_{a}=0u,\;\text{where}\;u∼N(\hat{\mu },\hat{\sigma })$$

Finally, the concatenated embedding is passed into the trained decoder to form a Gamma-Poisson distribution, from which the reconstructed gene expression profile matrix $\tilde{X}$ is sampled.


(12) $$\Lambda ∼\Gamma ({\hat{f}}_{\text{dec}}(Z_{b},Z_{p},Z_{a}),\Theta ,L)$$

(13) $$\tilde{X}∼P(\Lambda )$$

Formally, the joint probability distribution over the observed and latent variables is defined as:


(14) $$\begin{array}{cc} & p(X,Z_{b},M,E,U|P,A;\theta )=\left(\prod_{t=1}^{T}p(m_{t})p(e_{t})\right)p(u)\\ & \;\times \left(\prod_{n=1}^{N}p(z_{b,n})p(x_{n}|z_{b,n},p_{n},a_{n},M,E,U;\theta )\right) \end{array}$$

#### 2.2.5 GRN-aligned parameter optimization

As our work’s core idea is modeling explainable perturbation responses, we present several modifications implemented in GPO-VAE’s Latent Perturbation Encoder. Recall that the Latent Perturbation Encoder randomly samples sparse latent offsets using its inherent Bernoulli distribution sampler with trainable parameters $\hat{W}$. In CRADLE-VAE and SAMS-VAE, $\hat{W}\in R^{|G^{{}^{\circ}}|\times d}$, where the row vectors of the parameter matrix correspond to individual gene perturbation treatments. This design approach treats the sparse latent offsets as independent assumptions for each specific gene perturbation, enabling the model to account for distinct effects perturbation-wise.

**Figure 2.**
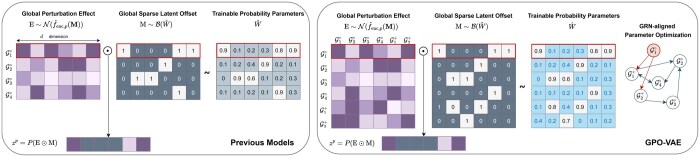
Comparison of perturbation encoder between previous models and our model. Unlike previous models using randomly sampled sparse latent offsets with trainable parameters, our model utilizes GRN-aligned parameter optimization for explainability.

We propose a redesigned approach for the Latent Perturbation Encoder. Specifically, we redefine ${\hat{W}}_{i,j}$ in $\hat{W}$ as the *causal probability* from source *i*th gene to target *j*th gene. Under this modified assumption, we reshape the parameters into a square matrix $\hat{W}\in R^{\left(|G^{{}^{\circ}}\cup G^{+}|)\times (|G^{{}^{\circ}}\cup G^{+}|\right)}$ including the extended gene subset. As illustrated in Fig. 2, this adjustment naturally enables interpretation of $\hat{W}$ as a gene regulatory network represented as an adjacency graph containing directed edge weights (i.e. causal gene-to-gene probabilities) and self-loops (i.e. self-regulating feedback mechanisms).

We then introduce GRN-based Parameter Optimization (GPO), a method that adjusts the parameters in $\hat{W}$ toward inclusion of topology-based model explainability to the latent perturbation effects. The loss objective from this method is composed by three components which are described as below.

First, we exploit the post-perturbational differential gene expression (DGE) values, which are calculated based on fold changes between control and perturbation gene expression profiles, as reference in adjusting the parameters. In detail, given ${\hat{W}}_{i,:}$ denotes the causal probabilities from source *i*th gene to all genes, each perturbation gene expression profile $x_{n}^{p}$ is paired with a control gene expression profile $x_{n}^{c}$ to build a reference differential gene expression profile $\Delta x_{n}$. For the pairing process, we used the Optimal Transport (OT) algorithm (Villani 2009). The reference differential gene expression profiles $\Delta X\in R^{N\times |G^{{}^{\circ}}\cup G^{+}|}$ are used to guide the trainable parameters in $\hat{W}$ through a loss function, which is mathematically expressed as follows:


(15) $$J_{\mathrm{dge}}(\hat{W})=\sum ||P\hat{W}-\Delta X|{|}_{1}$$

(16) $$\Delta x_{n}=\{\begin{array}{c} \text{Pairing}(x_{n}^{p},x_{n}^{c})\;\text{if}\;x_{n}^{c}\neq 0\\ 0\;\text{otherwise} \end{array}$$

Note that $x_{n}^{c}$ being a zero vector indicates a control sample excluded from constructing reference differential profiles. Importantly, all reference profiles used in this optimization are derived solely from the training dataset. We denote this component as **DGE loss** ($J_{\mathrm{dge}}$).

While the above loss objective adjusts the Latent Perturbation Encoder’s parameter space for its Bernoulli distribution sampler, it does not take all latent gene perturbation effects into account. In other words, only the row vectors in $\hat{W}$ which correspond to perturbation gene subset $G^{{}^{\circ}}$ are influenced by the loss objective, leaving the other parameters related to extended gene perturbation treatments ($G^{+}$) *not updated*.

Our key idea is to guide the optimization trajectories of GPO-VAE toward leveraging accumulated multi-hop causal relationships. As $\hat{W}$ is treated as a weighted adjacency matrix describing GRN, we can say that ${\hat{W}}^{k}$ contains *k*-hop causal relationships that explain the underlying biological mechanisms behind each gene perturbation. These *k*-hop causal relationships, which include extended genes as intermediate nodes, may contribute to expansive formation of the GRN by incrementally accumulating gene-gene relationships across different numbers of hops.

Given the reference differential gene expression profiles, we make the following modifications to $J_{\mathrm{dge}}$ as,


(17) $$J_{\mathrm{dge}}^{K}(\hat{W})=\sum ||P{\hat{T}}_{K}-\Delta X|{|}_{1}$$

(18) $${\hat{T}}_{K}=\hat{W}+\sum_{k=2}^{K}\frac{1}{\left|G^{{}^{\circ}}\cup G^{+}\right|}{\hat{W}}^{k}$$

We denote this component as **K-hop accumulation** ($J_{\mathrm{dge}}^{K}$) where *K* is equally set to 5 across all model configurations used in our experiments.

However, the current loss objective may increase the overall magnitude of values within ${\hat{T}}_{K}$ as the number of hops increases. This may cause proliferation of high causal probability values, leading to higher number of predicted interactions within the GRN that may be insignificant in terms of explainability.

To mitigate this issue, we penalize the parameters in $\hat{W}$ toward overall sparsity. Having adopted this approach from the NOTEARS algorithm (Zheng *et al.* 2018), we expect that only gene-gene interactions that are highly relevant with observed perturbation effects are retained, effectively reducing irrelevant ones. This refinement not only provides the model with more accurate information but also enhances overall predictive accuracy and explainability (Xu *et al.* 2022).

By combining the abovementioned three components, our proposed GPO loss objective is mathematically expressed as follows,


(19) $$J_{\mathrm{gpo}}(\hat{W})=J_{\mathrm{dge}}^{K}+J_{sp}$$

(20) $$=\sum ||P{\hat{T}}_{K}-\Delta X|{|}_{1}+||\hat{W}|{|}_{1}$$

where $J_{sp}$ is the added component denoted as **sparsity penalty**. The total loss objective for training GPO-VAE comprises maximization of the evidence lower bound (ELBO) ($J_{\mathrm{rec}}$, reconstruction of gene expression profiles), minimization of artifact disentanglement loss ($J_{\mathrm{ade}}$) and minimization of the newly proposed GPO loss objective ($J_{\mathrm{gpo}}$). The total loss objective (J) is mathematically expressed as follows,


(21) $$J(\phi ,\theta )=J_{\mathrm{rec}}(\theta ,\phi )+\alpha J_{\mathrm{ade}}(\phi )+\beta J_{\mathrm{gpo}}(\hat{W})$$

where ϕ and θ are the trainable parameters in GPO-VAE’s Encoder and Decoder modules respectively. Note that $\hat{W}\subset \phi$ as the Bernoulli distribution sampler parameterized by $\hat{W}$ is the component of the Latent Perturbation Encoder.

## 3 Experiments

### 3.1 Evaluation on perturbation response prediction

We conducted experiments to compare GPO-VAE’s performance on the gene perturbation response prediction task with other VAE-based models as baselines. The baseline VAEs used in the experiments are Conditional VAE (Sohn *et al.* 2015), sVAE+ (Lopez *et al.* 2023), SAMS-VAE (Bereket and Karaletsos 2024), CPA-VAE (Bereket and Karaletsos 2024), and CRADLE-VAE (Baek *et al.* 2025). Table 2 summarizes the key characteristics of each VAE-variant including GPO-VAE and its ablations. Details on each baseline are available in Supplementary Material S3.1.

**Table 2.** List of models used in gene perturbation response prediction experiments.

	GRN inference	Artifact disentanglement	Basal state as random variable	Sparsity assumption
Conditional VAE	×	×	×	×
SVAE+	×	×	×	√
SAMS-VAE	×	√	√	√
CPA-VAE	×	×	√	×
CRADLE-VAE	×	√	√	√
GPO-VAE (ours)	√	√	√	√

The evaluation metrics used in the experiments are Average Treatment Effect Pearson Correlation (**ATE-**ρ), Average Treatment Effect R-Square Score (**ATE-**$R^{2}$) (Bereket and Karaletsos 2024), and Jaccard Similarity between top 50 model-predicted and true differentially expressed genes (**Jaccard**) (Baek *et al.* 2025). The first two metrics compare differential expression values predicted by the model and the experimental data across all genes by calculating Pearson correlation coefficient and R-square respectively. Jaccard Similarity shows whether the predicted set of DEGs actually match the true set. Details on the evaluation metrics are available in Supplementary Material S4.

### 3.2 Evaluation on gene regulatory network inference

While GPO-VAE’s primary role is predicting the transcriptional outcomes of gene perturbations, our work focuses on aligning the optimization of $\hat{W}$ in its Latent Perturbation Encoder toward GRN-based explainability. To quantitatively evaluate the GRNs inferred by GPO-VAE based on its statistical explainability, we conducted additional experiments on GRN inference baseline methods.

The evaluation procedures, metrics and baseline methods for these experiments were adopted from CausalBench, a benchmark framework for evaluating GRN inference methods on single-cell gene perturbation datasets (Chevalley *et al.* 2025). The baseline methods used in the experiments are categorized based on type of data used for training: observational models use only observational data, while interventional models use both observational and interventional data. Observational models include PC (Spirtes *et al.* 2001), GES (Chickering 2002), Sortnregress (Reisach *et al.* 2021), GRNBoost (Aibar *et al.* 2017), NOTEARS (Linear), and NOTEARS(Linear, L1) (Zheng *et al.* 2018). Interventional models consist of DCDI-DSF (Brouillard *et al.* 2020), DCDI-G (Brouillard *et al.* 2020), GIES (Hauser and Bühlmann 2012), and DCDFG-MLP (Lopez *et al.* 2022). Details on each baseline are available in Supplementary Material S3.2.

While the baseline methods featured in CausalBench directly generate the inferred networks as output, GPO-VAE requires extraction of the optimized parameters from the Bernoulli distribution sampler within its Latent Perturbation Encoder. Note that each (*i*, *j*)th element in our Bernoulli parameter matrix $\hat{W}$ denotes the causal probability from the *i*th gene to *j*th gene. Since the parameters are scalar values ranging from 0 to 1, we determine the presence of edges by applying a threshold of 0.5. For a fair comparison with the baseline methods, we exclude the extended genes $G^{+}$ and include only the perturbed genes $G^{{}^{\circ}}$ in the evaluation.

We adopted statistical evaluation frameworks from CausalBench to evaluate the model’s capability of inferring GRNs that best align with gene perturbation data. Two evaluation metrics, Mean Wasserstein Distance (μWD) and False Omission Rate (**FOR**) were used in these experiments. Due to the absence of ground truth, they rely on the assumption that a predicted gene-gene interaction A to B should align with the statistical effects measured by expression changes of gene B when perturbing gene A. Specifically, μWD measures the average strength of causal effects of the inferred edges. For edge from A to B, WD is computed between the empirical distribution of the expression of B in control samples and in A-perturbed samples. A high μWD indicates stronger causal effects on the target node imposed by the source node. **FOR** measures the proportion of inferred negative edges that are statistically significant false negatives. Additional details are available in Supplementary Material S4.

### 3.3 Quantitative experimental results

As shown in Table 3, GPO-VAE outperforms the baseline models in predicting post-perturbation gene expression across all datasets. We attribute the performance improvement to the explicit modelling of GRN in the latent space. To further evaluate the robustness of the GRNs inferred by GPO-VAE, we conducted additional experiments, with the results shown in Table 4. We demonstrate that the GRN inferred by GPO-VAE achieves the best μWD and **FOR**, despite a trade-off observed among other baseline models for these metrics. In fact, increasing the number of edges in the predicted GRN typically reduces **FOR** by decreasing the total negative edges, but it can lead to poorer performance in terms of μWD due to the inclusion of more predicted positive edges. Remarkably, GPO-VAE excels in both metrics while maintaining a sufficient number of edges compared to other models. This underscores GPO-VAE’s ability to construct a sparse, data-consistent causal graph for the GRN, which in turn enhances its capacity to predict perturbation responses while also improving model explainability.

**Table 3.** Quantitative evaluation on Replogle K562, Replogle RPE1, Adamson dataset for Gene Perturbation Response Prediction.^a^

Dataset	Replogle K562			Replogle RPE1			Adamson		
Model/method	ATE-ρ ↑	ATE-$R^{2}$ ↑	Jaccard ↑	ATE-ρ ↑	ATE-$R^{2}$ ↑	Jaccard↑	ATE-ρ ↑	ATE-$R^{2}$ ↑	Jaccard ↑
Conditional VAE	0.6440 ± 0.00	0.4048 ± 0.00	0.2692 ± 0.00	0.6297 ± 0.01	0.3926 ± 0.01	0.3042 ± 0.00	0.7176 ± 0.02	0.4983 ± 0.02	0.3619 ± 0.01
SVAE+	0.4300 ± 0.01	0.1176 ± 0.02	0.1356 ± 0.00	0.5317 ± 0.03	0.2803 ± 0.04	0.1820 ± 0.01	0.6575 ± 0.02	0.4287 ± 0.03	0.2278 ± 0.01
SAMS-VAE	0.2093 ± 0.04	−0.3172 ± 0.06	0.0788 ± 0.01	0.1851 ± 0.04	−0.0650 ± 0.05	0.1188 ± 0.01	0.5741 ± 0.02	0.3034 ± 0.03	0.2031 ± 0.01
CPA-VAE	0.5740 ± 0.02	0.3283 ± 0.02	0.1960 ± 0.01	0.3944 ± 0.02	0.1463 ± 0.02	0.1710 ± 0.00	0.7585 ± 0.01	0.5686 ± 0.01	0.3240 ± 0.01
CRADLE-VAE	0.7373 ± 0.00	0.5358 ± 0.01	0.3095 ± 0.01	0.6248 ± 0.03	0.3814 ± 0.03	0.1947 ± 0.02	0.8549 ± 0.00	0.7189 ± 0.01	0.4067 ± 0.01
GPO-VAE	**0.7658** ± **0.00**	**0.5699** ± **0.01**	**0.3220** ± **0.01**	**0.6507** ± **0.02**	**0.4022** ± **0.03**	**0.2159** ± **0.02**	**0.8639** ± **0.00**	**0.7307** ± **0.01**	**0.4138** ± **0.01**

a The best performing results are highlighted in bold, while the second-best results are underlined.

**Table 4.** Quantitative evaluation on Replogle K562, Replogle RPE1, Adamson dataset for GRN inference.^a^

Dataset	Replogle K562			Replogle RPE1			Adamson		
Model/method	μ WD ↑	FOR ↓	# of Edges	μ WD ↑	FOR ↓	# of Edges	μ WD ↑	FOR ↓	# of Edges
Random1000	0.114 ± 0.001	0.176 ± 0.013	1000.0 ± 0.0	0.096 ± 0.003	0.130 ± 0.017	1000.0 ± 0.0	0.080 ± 0.013	0.236 ± 0.015	100.0 ± 0.0
Random10000	0.113 ± 0.001	−1.000 ± 0.000	10000.0 ± 0.0	0.098 ± 0.001	−1.000 ± 0.000	10000.0 ± 0.0	0.080 ± 0.004	−1.000 ± 0.000	1000.0 ± 0.0
PC	0.154 ± 0.002	0.188 ± 0.003	1598.6 ± 17.3	0.166 ± 0.003	0.150 ± 0.002	701.2 ± 8.4	0.115 ± 0.005	0.228 ± 0.022	210.0 ± 0.0
GES	0.160 ± 0.007	0.189 ± 0.003	636.8 ± 20.0	0.148 ± 0.009	0.153 ± 0.002	338.2 ± 9.3	0.134 ± 0.001	0.231 ± 0.019	88.0 ± 0.0
Sortnregress	0.161 ± 0.000	0.172 ± 0.000	5998.0 ± 0.0	0.159 ± 0.006	0.111 ± 0.002	2616.8 ± 47.4	0.127 ± 0.000	0.204 ± 0.000	236.0 ± 0.0
GRNBoost	0.132 ± 0.000	0.134 ± 0.000	150372.6 ± 829.8	0.123 ± 0.000	0.100 ± 0.000	59818.2 ± 438.0	0.086 ± 0.001	0.154 ± 0.007	2288.6 ± 60.8
NOTEARS (Linear)	0.164 ± 0.000	0.192 ± 0.000	8.0 ± 0.0	0.164 ± 0.000	0.147 ± 0.015	8.0 ± 0.0	0.000 ± 0.000	0.240 ± 0.020	0.0 ± 0.0
NOTEARS (Linear, L1)	0.140 ± 0.000	0.192 ± 0.000	6.0 ± 0.0	0.164 ± 0.000	0.154 ± 0.000	8.0 ± 0.0	0.000 ± 0.000	0.240 ± 0.020	0.0 ± 0.0
DCDI-DSF	0.162 ± 0.001	0.185 ± 0.002	4016.6 ± 38.2	0.166 ± 0.002	0.137 ± 0.003	1897.4 ± 97.1	0.110 ± 0.002	0.167 ± 0.003	537.8 ± 29.0
DCDI-G	0.184 ± 0.002	0.186 ± 0.004	2125.2 ± 39.7	0.182 ± 0.008	0.140 ± 0.003	946.8 ± 36.0	0.138 ± 0.001	0.211 ± 0.003	139.4 ± 8.0
GIES	0.155 ± 0.002	0.188 ± 0.003	1547.0 ± 36.5	0.144 ± 0.003	0.151 ± 0.003	717.4 ± 82.1	0.104 ± 0.011	0.236 ± 0.022	77.0 ± 6.0
DCDFG-MLP	0.137 ± 0.005	0.271 ± 0.184	9156.2 ± 2946.9	0.129 ± 0.012	0.136 ± 0.007	8696.8 ± 4004.9	0.079 ± 0.011	0.251 ± 0.019	494.4 ± 346.8
GPO-VAE	**0.248** ± **0.012**	**0.022** ± **0.007**	2887.8 ± 64.9	**0.280** ± **0.010**	**0.042** ± **0.008**	2211.4 ± 217.9	**0.159** ± **0.018**	**0.014** ± **0.005**	11.0 ± 2.6

a The best performing results are highlighted in bold, while the second-best results are underlined.

### 3.4 GRN topology analysis and ablation study

**Figure 3.**
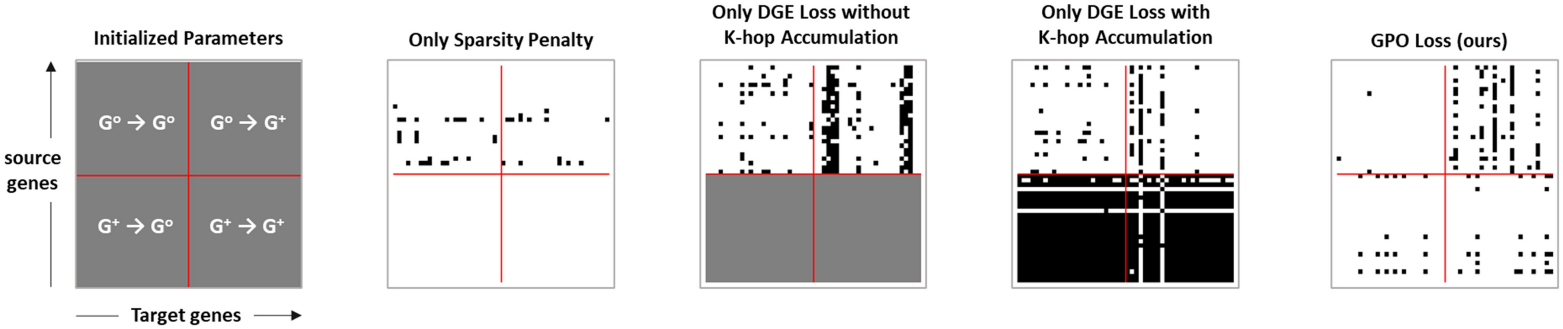
Adjacency matrices when different type/combination of loss objectives are applied. *X*-axes indicate target genes and *y*-axes indicate source genes $G^{{}^{\circ}}\cup G^{+}$. Red borderlines separate perturbation $G^{{}^{\circ}}$ and extended gene $G^{+}$ groups. The coloring scheme of the cells are based on the edge weights in each $\hat{W}$. White-colored, grey-colored, and black-colored cells denote “no edge” (<0.5), “initialized parameter” (=0.5), and “with edge” (>0.5), respectively.

To analyze the impact of our GRN-aligned parameter optimization, we conducted an ablation study and visualized the optimized parameters representing the directed, weighted adjacency matrix for the GRN during training in Fig. 3. Note that the parameters were extracted from $\hat{W}$ within the Latent Perturbation Encoder’s Bernoulli distribution sampler. Using the Replogle RPE1 dataset, we randomly sampled 25 perturbation genes and 25 extended genes for this analysis.

The ablations for GPO-VAE, related to the components that comprise our GPO loss objective, are the following—only sparsity penalty ($J_{sp}$), only DGE loss without K-hop accumulation ($J_{\mathrm{dge}}$) and only DGE loss with K-hop accumulation ($J_{\mathrm{dge}}^{K}$). We investigated the individual contributions of each component to the GRN-aligned optimization of $\hat{W}$.

Table 5 shows the results on ablation experiment while Fig. 3 illustrates the inferred GRN based on different ablation settings using heatmaps. Ablation model GPO-VAE ($J_{sp}$) suffered from a huge reduction in overall statistical significance of predicted edges, leading to poor μWD. Its inferred GRN exhibits edges exclusively originating from perturbation genes, with no edges involving extended genes. This outcome reflects the penalty’s tendency to suppress connections related to extended genes, as their parameters were never optimized.

**Table 5.** Results on ablation experiments of GPO-VAE.^a^

Model	ATE-ρ ↑	ATE-$R^{2}$ ↑	Jaccard ↑	μ WD ↑	FOR ↓	No. of edges
GPO-VAE ($J_{sp}$)	0.6416 ± 0.01	0.3904 ± 0.01	0.2099 ± 0.01	0.115 ± 0.004	0.040 ± 0.007	5516.4 ± 111.6
GPO-VAE ($J_{\mathrm{dge}}$)	0.6435 ± 0.02	0.3939 ± 0.02	0.2074 ± 0.01	0.192 ± 0.001	0.040 ± 0.011	7970.8 ± 168.7
GPO-VAE ($J_{\mathrm{dge}}^{K}$)	**0.6593** ± **0.01**	0.4146 ± 0.01	0.2197 ± 0.01	0.157 ± 0.000	0.042 ± 0.005	16034.2 ± 315.3
GPO-VAE ($J_{\mathrm{gpo}}$, ours)	0.6584 ± 0.02	**0.4169** ± **0.02**	**0.2215** ± **0.01**	**0.414** ± **0.010**	**0.039** ± **0.007**	854.8 ± 48.1

a The best performing results are highlighted in bold, while the second-best results are underlined.

Conversely, ablation model GPO-VAE ($J_{\mathrm{dge}}$) constructed a GRN with relatively less sparsity and higher μWD, compared to the ablated model using only sparsity penalty. However, all inferred edges containing extended genes maintained uniform weights of 0.5 throughout model training, indicating that these relationships were never been accounted during optimization.

The GRN optimized with $J_{\mathrm{dge}}^{K}$ which has no sparsity regularization, demonstrates the involvement of both perturbed and extended genes in the optimization process. This suggests that extended genes contributing novel pathways to perturbation gene relationships were effectively optimized. However, its inferred GRN remains dense, particularly in the edges involving extended genes, decreasing μWD.

Finally, the GRN inferred by our model using the GPO loss objective $J_{\mathrm{gpo}}$ is both sparse and demonstrates well-optimized relationships involving extended genes, as shown in Fig. 3. The balance between the effects of sparsity penalty and multi-hop causal relationships contributed to GPO-VAE outperforming its ablations in terms of GRN inference along with a slight improvement in perturbation response prediction.

### 3.5 Biological implication of the inferred GRN

To investigate the impact of involving the extended gene subset in GRN inference on establishing meaningful gene-gene causal relationships, we studied the extracted subnetworks from Replogle K562 dataset, both biologically and statistically. We hypothesize that novel pathways created from the interaction of extended genes would provide biologically relevant insights related to cancer progression or immune system disorders in K562 cells, derived from a chronic myelogenous leukemia patient. Subnetworks were selected based on two criteria: (i) paths with perturbed genes connected via at least one extended gene, and (ii) no directed edge between the selected perturbed genes in the GRN inferred by DCDI-G, the best baseline model. The newly formed 2- or 3-hop causal edges exhibit high WDs, indicating strong causal effects. We then analyzed the gene sets within the subnetworks using DAVID Functional Annotation Tool.

**Figure 4.**
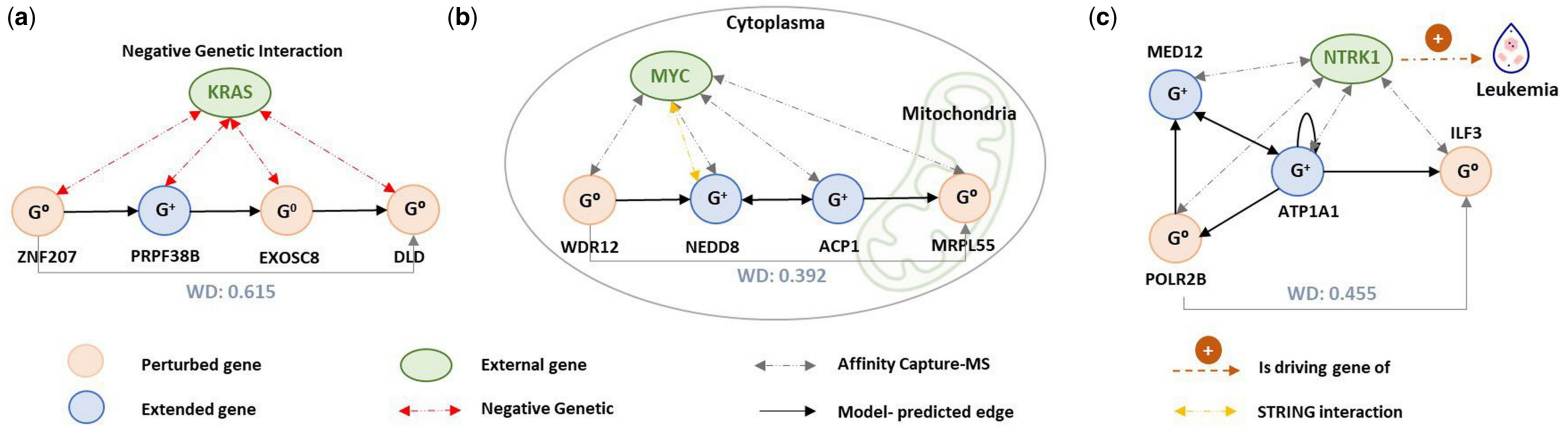
Case study and pathway analysis of GRN subnetworks involved in the interaction with three cancer proteins: KRAS, Myc, NTRK1. Solid lines indicate model-inferred edges (black: one-hop edge; grey: 2- or 3-hop edge); dotted lines indicate literature-proven pathways.

As shown in Fig. 4, analysis revealed that genes (or proteins) in the connected subnetworks interact with KRAS, MYC, and NTRK1, all of which are well-known cancer-associated genes (proteins), respectively. This finding highlights the consistency between the GPO-VAE-inferred GRN and prior biological knowledge.

In the KRAS-related subnetwork shown in Fig. 4a, all genes were experimentally confirmed to have a negative genetic relationship with KRAS (Vichas *et al.* 2021). In this context, negative genetic interactions occur when mutations in individual genes cause minimal phenotypic effects, but their combined mutation in the same cell results in a severe fitness defect or lethality. Interestingly, although STRING—a widely used biological network database—did not identify interactions between these genes, experimental evidence supports the validity of our subnetwork (Szklarczyk *et al.* 2023). This suggests that the inferred subnetwork could provide valuable insights for identifying new synthetic lethal gene pairs, potentially advancing target discovery efforts related to KRAS.

In Fig. 4b, the genes in the subnetwork is found to exhibit protein-level interaction with MYC, identified through mass spectrometric methods (Heidelberger *et al.* 2018, Solvie *et al.* 2022, Wang *et al.* 2022). Only interaction between MYC and NEDD8 is documented in STRING, highlighting the advantage of our extended subnetwork. By including extended genes such as NEDD8, which were not utilized in other baseline GRN inference methods, our approach expands the search space, enabling the discovery of additional pathways that align with prior biological networks. Moreover, leveraging the genotype-phenotype mapping derived from *Replogle et al.* we identified WDR12, NEDD8, and MRPL55 as genes associated with the ribosomal subunit. On the other hand, subcellular localization data from UniProtKB/Swiss-Prot revealed that MYC, WDR12, NEDD8, and ACP1 are highly likely to localize in the cytosol, while MRPL55 is localized in the mitochondrion. Notably, the Replogle paper focused on mitochondrial genome stress responses, and prior research links MYC to cancer-related phenotypes, such as cell cycle regulation and MYC-dependent apoptosis, through mitochondrial targets. MRPL55, in particular, was reported as a nuclear-encoded mitochondrial gene downstream target in this context (Morrish and Hockenbery 2014). These findings suggest that the inferred MYC-related subnetwork reflects a MYC-driven pathway that transitions from the cytosol to the mitochondria, contributing to cancer-related phenotypes linked to cell cycle and mitochondrial biogenesis.

The last subnetwork shown in Fig. 4c demonstrated protein-level interaction with NTRK1, an oncogenic driver discovered for leukemia studied in prior studies (Joshi *et al.* 2019). While STRING did not identify interactions among the genes in this subnetwork, our model revealed that MED12, ATP1A1, ILF3, and POLR2B could also serve as potential target genes for chronic myelogenous leukemia. Surprisingly, all genes in this subnetwork were associated with leukemia types, including acute myeloid leukemia, chronic lymphocytic leukemia, and chronic myelogenous leukemia as supported by prior studies (Kämpjärvi *et al.* 2014, Lal *et al.* 2017, Nazitto *et al.* 2021, Richter *et al.* 2022). This evidence suggests that the genes identified by our subnetwork could be promising candidates for discovering novel therapeutic targets for immune-related diseases.

These results indicate that GPO-VAE constructs biologically and statistically meaningful edges through GRN-aligned parameter optimization of extended genes.

### 3.6 Case study on unseen perturbation treatments

We hypothesize that by leveraging the ability of the GPO loss to optimize the causal probabilities of edges formed within a k-hop neighborhood, GPO-VAE should be capable of predicting gene expression for unseen perturbation treatments. To verify this, we excluded samples involving three distinct perturbation treatments from the training dataset and assessed the model’s performance in accurately predicting gene expression. We calculated μWD across all perturbed genes and identified the top three genes with the highest combined in-degree and out-degree, using a threshold of 0.3. These selected genes represent key hub genes within the network, as they both receive the most influence from other genes and exert the greatest influence on others.

**Figure 5.**
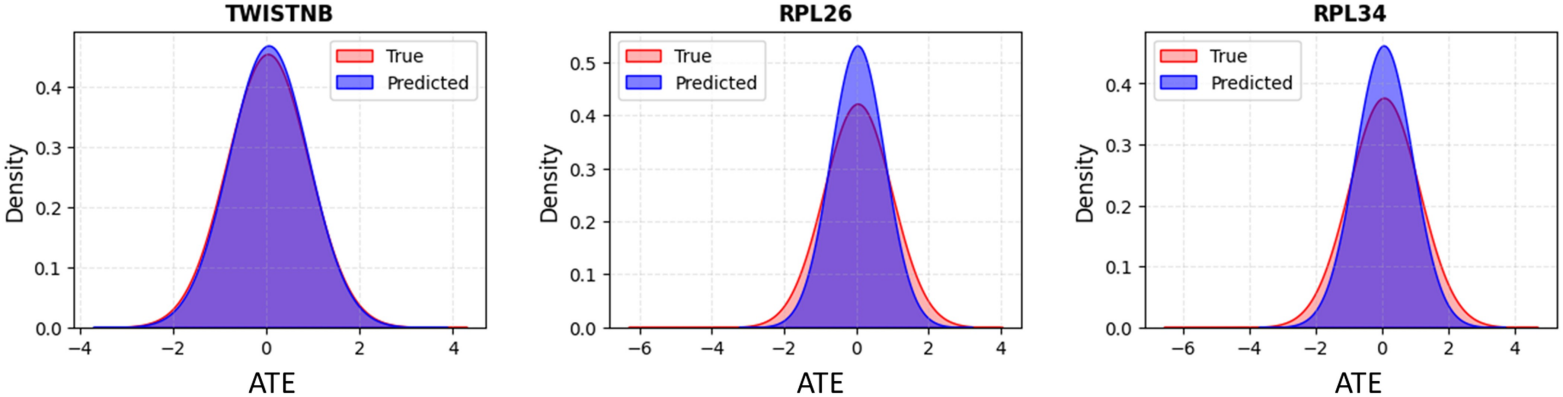
Quantitative performance on unseen perturbations for TWISTNB, RPL26, and RPL34, with *x*-axis showing scaled ATE and *y*-axis the probability density. For TWISTNB, the ATE Pearson correlation, ATE *R*², and Jaccard similarity are 0.792, 0.578, and 0.587, respectively. For RPL26, the corresponding values are 0.886, 0.775, and 0.724, and for RPL34, they are 0.919, 0.831, and 0.667.

GPO-VAE achieves competitive performance across all three excluded cases, with **ATE**-ρ of 0.79, 0.88, and 0.91 and **ATE**-$R^{2}$ scores of 0.57, 0.77, and 0.83. Additionally, as shown in Fig. 5, the scaled ATE distribution of predicted values align closely with that of actual values, demonstrating the model’s ability to approximate post-perturbation responses. These results highlight GPO-VAE’s ability to generalize to unseen perturbations, showcasing its robustness and scalability for gene expression prediction in practical applications.

## 4 Conclusion

In this work, we introduce GPO-VAE, a GRN-enhanced explainable VAE, that aligns the latent subspaces associated with gene-specific perturbation effects with gene-gene causal relationship for improved post-perturbation expression prediction. This is achieved through GRN-aligned parameter optimization, which refines the causal gene-gene relationships toward biologically meaningful GRN. Through evaluations on three benchmark datasets and case studies, we demonstrate that GPO-VAE not only accurately predicts cellular responses to gene perturbations but also constructs statistically robust and biologically relevant GRNs.

One limitation of GPO-VAE is that, unlike perturbation response prediction models such as CRADLE-VAE and SAMS-VAE, which have demonstrated their ability to predict responses to multi-gene perturbation treatments, our model has not yet been explicitly designed for such scenarios though the architecture itself does not inherently restrict its application to multi-gene perturbation settings.

Moreover, we identified a misalignment between the two evaluation schemes—biological and statistical—introduced in CausalBench. Furthermore, the reference networks proposed in biological evaluation schemes are not cell-type specific and may introduce biases. This observation highlights the inherent challenge of evaluating GRN inference models, as the absence of ground truth limits the comprehensiveness of existing evaluation methods.

As future work, we aim to extend GPO-VAE to incorporate multi-gene perturbation treatments and enable the model to leverage relationships such as synergy, inhibition, and synthetic lethality within gene regulatory networks for improved perturbation response prediction. Also, we plan to utilize prior biological knowledge, to construct a GRN that strongly aligns with both existing biological knowledge and perturbation experimental data.

## Supplementary Material

btaf256_Supplementary_Data

## Data Availability

Our study used open-access datasets, and preprocessed data-related links are available in the github repository mentioned above.

## References

[btaf256-B1] Adamson B , Norman TM, Jost M et al A multiplexed single-cell crispr screening platform enables systematic dissection of the unfolded protein response. Cell 2016;167:1867–82.e21. 27984733 10.1016/j.cell.2016.11.048 PMC5315571

[btaf256-B2] Aibar S , González-Blas CB, Moerman T et al Scenic: single-cell regulatory network inference and clustering. Nat Methods 2017;14:1083–6. 28991892 10.1038/nmeth.4463 PMC5937676

[btaf256-B3] Amann J , Blasimme A, Vayena E et al; Precise4Q Consortium. Explainability for artificial intelligence in healthcare: a multidisciplinary perspective. BMC Med Inf Decision Mak 2020;20:310–9. 10.1186/s12911-020-01332-6 PMC7706019 33256715

[btaf256-B4] Baek S , Park S, Chok YT et al Cradle-VAE: enhancing single-cell gene perturbation modeling with counterfactual reasoning-based artifact disentanglement. *AAAI* 2025;39:15445–52.

[btaf256-B5] Bereket M , Karaletsos T. Modelling cellular perturbations with the sparse additive mechanism shift variational autoencoder. Adv Neural Inf Process Syst 2024;36:1–12.

[btaf256-B6] Brouillard P , Lachapelle S, Lacoste A et al Differentiable causal discovery from interventional data. Adv Neural Inf Process Syst 2020;33:21865–77.

[btaf256-B7] Charte D , Charte F, del Jesus MJ et al An analysis on the use of autoencoders for representation learning: fundamentals, learning task case studies, explainability and challenges. Neurocomputing 2020;404:93–107.

[btaf256-B8] Chevalley M , Roohani Y, Mehrjou A et al A large-scale benchmark for network inference from single-cell perturbation data. *Commun Biol* 2025;8:412. 10.1038/s42003-025-07764-y PMC11897147 40069299

[btaf256-B9] Chickering DM. Optimal structure identification with greedy search. J Mach Learn Res 2002;3:507–54.

[btaf256-B10] Gavriilidis GI , Vasileiou V, Orfanou A et al A mini-review on perturbation modelling across single-cell omic modalities. Comput Struct Biotechnol J 2024;23:1886–96. 38721585 10.1016/j.csbj.2024.04.058 PMC11076269

[btaf256-B11] Hauser A , Bühlmann P. Characterization and greedy learning of interventional Markov equivalence classes of directed acyclic graphs. J Mach Learn Res 2012;13:2409–64.

[btaf256-B12] Heidelberger JB , Voigt A, Borisova ME et al Proteomic profiling of VCP substrates links VCP to k6-linked ubiquitylation and c-MYC function. EMBO Rep 2018;19:e44754. 29467282 10.15252/embr.201744754 PMC5891417

[btaf256-B13] Joshi SK , Davare MA, Druker BJ et al Revisiting NTRKS as an emerging oncogene in hematological malignancies. Leukemia 2019;33:2563–74. 31551508 10.1038/s41375-019-0576-8 PMC7410820

[btaf256-B14] Kämpjärvi K , Järvinen TM, Heikkinen T et al Somatic med12 mutations are associated with poor prognosis markers in chronic lymphocytic leukemia. Oncotarget 2014;6:1884–8. 10.18632/oncotarget.2753 PMC4359339 25595892

[btaf256-B15] Lal R , Lind K, Heitzer E et al Somatic tp53 mutations characterize preleukemic stem cells in acute myeloid leukemia. Blood J Am Soc Hematol 2017;129:2587–91. 10.1182/blood-2016-11-751008 28258055

[btaf256-B16] Lopez R , Hütter J-C, Pritchard J et al Large-scale differentiable causal discovery of factor graphs. Adv Neural Inf Process Syst 2022;35:19290–303.

[btaf256-B17] Lopez R , Tagasovska N, Ra S et al Learning causal representations of single cells via sparse mechanism shift modeling. In: *Conference on Causal Learning and Reasoning, Tübingen*. PMLR, 2023, 662–91.

[btaf256-B18] Morrish F , Hockenbery D. Myc and mitochondrial biogenesis. Cold Spring Harb Perspect Med 2014;4:a014225. 24789872 10.1101/cshperspect.a014225 PMC3996374

[btaf256-B19] Nazitto R , Amon LM, Mast FD et al Ilf3 is a negative transcriptional regulator of innate immune responses and myeloid dendritic cell maturation. J Immunol 2021;206:2949–65. 34031149 10.4049/jimmunol.2001235 PMC8611108

[btaf256-B20] Reisach A , Seiler C, Weichwald S. Beware of the simulated dag! causal discovery benchmarks may be easy to game. Adv Neural Inf Process Syst 2021;34:27772–84.

[btaf256-B21] Replogle JM , Norman TM, Xu A et al Combinatorial single-cell CRISPR screens by direct guide rna capture and targeted sequencing. Nat Biotechnol 2020;38:954–61. 32231336 10.1038/s41587-020-0470-y PMC7416462

[btaf256-B22] Replogle JM , Saunders RA, Pogson AN et al Mapping information-rich genotype-phenotype landscapes with genome-scale perturb-seq. Cell 2022;185:2559–75.e28. 35688146 10.1016/j.cell.2022.05.013 PMC9380471

[btaf256-B23] Richter A , Lange S, Holz C et al Effective tumor cell abrogation via venetoclax-mediated bcl-2 inhibition in kmt2a-rearranged acute b-lymphoblastic leukemia. Cell Death Discov 2022;8:302. 35778418 10.1038/s41420-022-01093-3 PMC9249764

[btaf256-B24] Schölkopf B , Locatello F, Bauer S et al Toward causal representation learning. Proc IEEE 2021;109:612–34.

[btaf256-B25] Sohn K , Lee H, Yan X. Learning structured output representation using deep conditional generative models. Adv Neural Inf Process Syst 2015;28:3483–8.

[btaf256-B26] Solvie D , Baluapuri A, Uhl L et al Myc multimers shield stalled replication forks from RNA polymerase. Nature 2022;612:148–55. 36424410 10.1038/s41586-022-05469-4

[btaf256-B27] Spirtes P , Glymour C, Scheines R. Causation, Prediction, and Search. Cambridge: MIT Press, 2001.

[btaf256-B28] Szklarczyk D , Kirsch R, Koutrouli M et al The string database in 2023: protein–protein association networks and functional enrichment analyses for any sequenced genome of interest. Nucleic Acids Res 2023;51:D638–46. 36370105 10.1093/nar/gkac1000 PMC9825434

[btaf256-B29] Vichas A , Riley AK, Nkinsi NT et al Integrative oncogene-dependency mapping identifies RIT1 vulnerabilities and synergies in lung cancer. Nat Commun 2021;12:4789. 34373451 10.1038/s41467-021-24841-y PMC8352964

[btaf256-B30] Villani C. *Optimal Transport: Old and New*, Vol. 338. Heidelberg: Springer, 2009.

[btaf256-B31] Wang L , Chen C, Song Z et al Ezh2 depletion potentiates myc degradation inhibiting neuroblastoma and small cell carcinoma tumor formation. Nat Commun 2022;13:12. 35013218 10.1038/s41467-021-27609-6 PMC8748958

[btaf256-B32] Xu D , Gao E, Huang W et al On the sparse dag structure learning based on adaptive lasso. arXiv, arXiv:2209.02946, 2022, preprint: not peer reviewed.

[btaf256-B33] Zheng X , Aragam B, Ravikumar PK et al Dags with no tears: continuous optimization for structure learning. Adv Neural Inf Process Syste 2018;31:12.

