# Supplementary material for: GPO-VAE: modeling explainable gene perturbation responses utilizing GRN-aligned parameter optimization
Source: Bioinformatics. 2025 Jul 15;41(Suppl 1):i599–608. doi: 10.1093/bioinformatics/btaf256 (PMC12261430; doi:10.1093/bioinformatics/btaf256)

Supplementary Material

S1. Quality Control (QC) Criteria

We adopted the six QC criteria defined in CRADLE-VAE(Baek et al, 2024), in line with 10X Genomics, for the QC annotation of each of the gene expression data instances. The QC criteria are as follows:

1. **UMI Counts**: Unique Molecular Identifiers (UMIs) represent the distinct molecular tags added to RNA molecules during the preparation phase of single-cell RNA sequencing (scRNA-seq). The total UMI count per cell indicates the number of unique RNA molecules detected. Filtering out cells with very low UMI counts can help reduce noise and improve data reliability.
2. **Number of Features**: This metric refers to the number of unique genes or transcripts identified in each cell. Filtering out cells with excessively high or low feature counts is essential to remove potential anomalies, such as multiplets or droplets contaminated with ambient RNA. A high feature count suggests that the cell is expressing a diverse set of genes, typically seen in viable and healthy cells. Conversely, cells with very few features are likely non-viable and are often excluded during preprocessing.
3. **Mitochondrial Read Percentage**: The proportion of RNA reads originating from mitochondrial genes is an indicator of cellular health. Cells with a high percentage of mitochondrial RNA are often stressed or damaged, making this metric a valuable filter during quality control.
4. **Hemoglobin Read Percentage**: In scRNA-seq, hemoglobin-associated transcripts reflect the activity of hemoglobin genes, primarily found in red blood cells. In experiments focusing on non-hematopoietic tissues, a high percentage of hemoglobin reads may indicate sample contamination or preparation issues.
5. **Ribosomal Read Percentage**: This refers to the fraction of sequencing reads derived from ribosomal RNA (rRNA). An unusually high proportion of ribosomal reads might suggest an inefficient rRNA depletion step, leading to less usable data for gene expression analysis as rRNA can dominate the sequencing output.
6. **Doublets**: Doublets occur when two or more cells are accidentally captured together in the same droplet or well during sequencing. These artifacts result in combined gene expression profiles that can mimic or distort real biological states, making it necessary to detect and exclude doublets to maintain data accuracy.

S2. Methods

S2.1 Preprocessing Details

The preprocessing procedure followed the standard guidelines provided by the CausalBench framework. The single-cell RNA sequencing (scRNA-seq) data preprocessing involved several quality-control and filtering steps to ensure reliable downstream analyses:

Identification of Strong Perturbations:

Dataset	Weak Perturbation
adamson	20
replogle rpe1	850
replogle K562	965

Genetic perturbations demonstrating a strong biological effect were selected based on predefined statistical criteria. Perturbations were classified as 'strong' if they met the following conditions:

- Number of Differentially Expressed Genes (DEGs), determined using the Anderson-Darling test, was greater than 50.
- Perturbation efficiency, measured as percent knockdown, was at least 30% (i.e., ≤ -0.3).
- The number of associated cells was greater than 25.

Filtering Cells by Perturbation Effect:
Cells were further refined based on the effect of each perturbation. For each gene perturbation, we identified the bottom 10th percentile of expression levels observed in control (non-targeting) cells for the corresponding perturbed gene. Only perturbed cells exhibiting gene expression below this threshold were retained, ensuring that the analyzed cells reflect meaningful perturbation effects.

Filtering Cells by Data Availability:
Perturbations associated with insufficient numbers of cells were excluded from further analysis. Specifically, perturbations with fewer than 100 cells were removed to maintain statistical robustness.

These preprocessing steps collectively ensured the robustness and relevance of the dataset for subsequent causal discovery analyses.

S2.2 Counterfactual Reasoning

Counterfactual reasoning in machine learning explores hypothetical scenarios by estimating potential outcomes under alternative conditions. This approach addresses the question: "What would the model predict if a different action had been taken?" In the context of causal relationships, counterfactual analysis is particularly valuable for understanding the impact of various treatments or interventions. In this study, we employ counterfactual reasoning to investigate a specific question: "*What would the outcome have been if the result had not been influenced by technical artifacts, given a particular treatment (perturbation)*?"

Following CRADLE-VAE, we employed counterfactual reasoning-based artifact disentanglement in the latent artifact encoder module. Latent Artifact Encoder models the distribution of technical artifacts independently of basal cell features and perturbations. During training, it samples a global latent artifact embedding $\mathbf{u} \in \mathbb{R}^{1\times d}$ from a parameterized Normal distribution $\mathcal{N}(\hat{\mu}, \hat{\sigma})$. The sampled embedding $\mathbf{Z}_a \in n \times d$, which represents a technical artifact, is then masked if the input gene expression profile is annotated as artifact-free with a QC label of 0 (QC passed), otherwise it is retained ($A \in \{0,1\}^{n\times 1}$). In addition, it creates a counterfactual $\mathbf{Z}_{a,c} \in n \times d$ of the global artifact embedding by applying the opposite masking operation $(1 - A)$ (i.e., retaining the embedding if it was originally zeroed and vice versa).

The Latent Artifact Encoder that takes the QC labels of n data instances as input ($A \in \{0,1\}^{n\times 1}$) is mathematically expressed as follows,

$$\mathbf{u} \sim \mathcal{N}(\hat{\mu}, \hat{\sigma})$$
$$\mathbf{Z}_a = A\mathbf{u}$$
$$\mathbf{Z}_{a,c} = (1 - A)\mathbf{u}$$

where,

- $\mathbf{u} \in \mathbb{R}^{1\times d}$ is the global latent artifact embedding.
- $\mathcal{N}(\hat{\mu}, \hat{\sigma})$ is the Normal Distribution sampler with trainable parameters
- $\mathbf{Z}_a \in n \times d$ is the latent artifact embedding matrix for the n data instances.

- $\mathbf{Z}_{a,c} \in n \times d$ is the counterfactual latent artifact embedding matrix for the n data instances.

Meanwhile, the Latent Basal State Encoder that takes the gene expression profiles of n data instances as input ($X \in \{0,1\}^{n \times |\mathcal{G}}$ is mathematically expressed as follows,

$$\mathbf{Z}_{b,c} \sim \mathcal{N}(\hat{f}_{(\text{enc},\mu)}(\mathbf{X}, \mathbf{Z}_p, \mathbf{Z}_{a,c}), \hat{f}_{(\text{enc},\sigma)}(\mathbf{X}, \mathbf{Z}_p, \mathbf{Z}_{a,c}))$$

$$\bar{\mathbf{Z}}_{b,c} \sim \mathcal{N}(\hat{f}_{(\text{enc},\mu)}(\bar{\mathbf{X}}, \mathbf{Z}_p, \mathbf{Z}_{a,c})\hat{f}_{(\text{enc},\sigma)}(\bar{\mathbf{X}}, \mathbf{Z}_p, \mathbf{Z}_{a,c}))$$

$$\mathbf{Z}_b \sim \mathcal{N}(\hat{f}_{(\text{enc},\mu)}(\mathbf{X}, \mathbf{Z}_p, \mathbf{Z}_a), \hat{f}_{(\text{enc},\sigma)}(\mathbf{X}, \mathbf{Z}_p, \mathbf{Z}_a))$$

where,

- $\hat{f}_{(\text{enc},\mu)}, \hat{f}_{(\text{enc},\sigma)}$ are the encoder-specialized neural networks that output the mean, variance parameters for the Normal Distribution sampler $\mathcal{N}(\cdot,\cdot)$ respectively.
- $\mathbf{Z}_{b,c} \in \mathbb{R}^{n \times d}$ is the counterfactual latent basal state embedding matrix for the n data instances.
- $\bar{\mathbf{Z}}_{b,c} \in \mathbb{R}^{n \times d}$ is the reference counterfactual latent basal state embedding matrix for the n data instances.
- $\mathbf{Z}_b \in \mathbb{R}^{n \times d}$ is the latent basal state embedding matrix for the n data instances.

Here, an auxiliary loss objective is imposed to guide $\mathbf{z}^b_{i,c}$ to align with $\bar{\mathbf{z}}^b_{i,c}$. Introduced in Baek et al., this is done by minimizing the KL divergence between the latent basal state embeddings between counterfactual latent basal state embedding and the reference counterfactual latent basal state embedding. The auxiliary loss is formally defined as:

$$\mathcal{J}_2(\phi) = -\text{KL}\left[q(Z^b_c|X, P, A; \phi) \| q(\bar{Z}^b_c|\bar{X}, P, A; \phi)\right]$$

S2.3 Optimal Transport

We employed optimal transport in sample pairing between interventional data observational data to calculate ΔX for optimization of GRN using DGE loss. Optimal Transport (OT) is commonly employed to assess the similarity between distributions, particularly when their supports do not overlap. In cases where the supports are disjoint, OT-based Wasserstein distances offer advantages over widely used f-divergences, such as the Kullback-Leibler divergence, Jensen-Shannon divergence, and Total Variation distance. The objective of OT is as below:

$$\min_{m, m\#\mu_s = \mu_t} \int c(x, m(x)) d\mu_s(x)$$

where $c(\cdot,\cdot)$ is the ground cost.

For implementation details, refer to https://pythonot.github.io/

S3. Baselines

S3.1 VAE-based Perturbation Response Prediction Models

Below includes brief descriptions of the baselines we employed in our experiments.

Conditional VAE
Conditional VAE, first introduced for structured output prediction tasks, is a deep generative model that utilizes stochastic neural networks with Gaussian latent variables. It incorporates features including a VAE backbone and input omission noise, which helps regularize the deep neural network during the training process.

SVAE+
In line with the sparse mechanism shift hypothesis, SVAE+ tackles data sparsity explicitly through a masking and embedding mechanism. However, different from other VAE-based baseline models, the model architecture itself is not designed for modelling the treatment effect of multiple interventions. Rather, it generates a cell's complete latent embedding by sampling from a learned prior conditioned on the treatment applied to the cell.

SAMS-VAE
SAMS-VAE is a VAE-based generative model designed for modelling cellular perturbation effect. It incorporates sparsity in the latent global perturbation variables to learn disentangled, pertrubation-specific latent subspace and the latent basal state. It extends SVAE+ by capturing interventions and their sparse effects as explicit latent additive latent variables.

CPA-VAE
CPA-VAE is an ablated version of SAMS-VAE defined by Bereket and Karaletsos, where all mask components are fixed to 1. In other words, it excludes sparsity from the latent perturbation effects. However, it retains the advantages of the improved inference methods used for correlated variational families.

CRADLE-VAE
CRADLE-VAE addresses the issue of unwanted artifacts effect present in data by utilizing the quality control (QC) annotation, and thereby increasing the number of gene expression data available for training. It adopts the architecture of SAMS-VAE while additionally incorporating a latent artifact encoder for artifacts effect distribution modelling. Our model inherits the basic architecture and concepts in CRADLE-VAE but increases the explainability of the latent subspaces by aligning it to gene regulatory networks via GRN loss.

S3.2 Gene Regulatory Network Inference Methods
We adopted the baselines for both of which leverages observational and interventional data, as introduced in CausalBench challenges.

PC
Though not specifically designed for gene regulatory network inference, this method is among the most commonly employed approaches for causal inference using observational data. It relies on the assumption of no confounding variables and determines conditional independence to provide results that are asymptotically accurate. The output is an equivalence class of graphs that align with the outcomes of the conditional independence tests.

Greedy Equivalence Search (GES)
GES is a general score-based algorithm for inferring causal structures from observational data. This method uses a two-phase process, consisting of Forward and Backward phases, to iteratively add and remove edges from the graph while computing a score to select within an equivalence class.

SORTnREGRESS

Sortnregress is a machine learning-based method specifically developed for Gene Regulatory Network (GRN) inference, focusing on ranking and regression to identify regulatory relationships. The method evaluates candidate parent genes for each target gene by their ability to predict the expression levels of the downstream gene.

GRNBoost
Unlike other algorithms above, GRNBoost is a method specifically designed for Gene Regulatory Network (GRN) inference, leveraging Gradient Boosting tree models to identify regulatory relationships. GRNBoost operates by evaluating candidate parent genes for each target gene, ranking them based on their ability to predict the expression profile of the downstream gene. This ranking serves as a feature selection process, helping to identify the most relevant interactions and infer the underlying network structure.

NOTEARS
This approach reformulates the problem of inferring a DAG into a continuous optimization task over real-valued matrices, bypassing the need for combinatorial searches across acyclic graphs. It accomplishes this by designing a smooth function, with easily computable derivatives, that equals zero only when the corresponding graph is acyclic. Different variants of NOTEARS are defined by the type of regularization term included in the loss function (e.g., L1 regularization to enforce sparsity).

Differentiable Causal Discovery with Interventions (DCDI)
DCDI is a framework that combines neural networks and gradient-based optimization techniques to infer Directed Acyclic Graphs (DAGs) by leveraging interventional data. It is specifically designed to handle various types of interventions, including perfect interventions (where the target variable is completely determined), imperfect interventions (where the target variable retains some level of uncertainty), and unknown interventions (where the target of intervention is not explicitly identified). Variants of DCDI include **DCDI-G** which assumes that the conditional distributions of variables given their parents are Gaussian, **DCDI-DSF** which removes the Gaussian assumption and utilizes normalizing flows to model more complex and flexible conditional distribution.

Greedy Interventional Equivalence Search (GIES)
GIES extends GES to incorporate interventional data, which provides additional information about causal relationships. Interventional data is generated when certain variables are experimentally manipulated, breaking natural dependencies and revealing causal directions. To leverage intervention data, GIES adds a third phase, called the "turning phase," which adjusts the orientation of edges based on interventional data.

Random (k)
Random (k) serves as the simplest baseline, generating a graph by randomly selecting k nodes uniformly without replacement. In our experiments, we evaluated the approach using k=1000.

S4. Evaluation Metrics

Average Treatment Effect-Pearson (ATE-Pearson) & -R^2 (ATE-R^2)
As employed by Bereket and Karaletsos, ATE-Pearson and ATE-R^2 is a metric that measures the correlation between the average treatment effect of the predicted expression and the average differential expression (DE) of the real data. In our experiments, we estimate the model average treatment effect with K = 2,500 particles for reporting the results.

Jaccard Top k
Jaccard Top k measures the ability of a model in accurately predicting the top k differentially expressed genes given a type of perturbation. Specifically, we calculate the Jaccard Index between the set of top-k differentially expressed genes predicted by the model and the one from the real data.

Mean Wasserstein Distance (µWD)
Mean Wasserstein Distance (µWD) measures the average strength of causal effects of the inferred edges. For edge from A to B, Wasserstein distance is computed between the empirical distribution of the expression of B in control samples and in A-perturbed samples. Then the average of all inferred edges is calculated. A high µWD indicates stronger causal effects on the child imposed by the parent.

False Omission Rate (FOR)
FOR measures the proportion of False Negatives among the inferred Negatives. Due to the absence of ground truth graph, false negatives are determined by performing two-sided Mann–Whitney U rank test between samples from the expression of B in control samples and in A-perturbed samples for all sampled negative pairs. An edge is deemed false negative if the null hypothesis that the two distributions are equal is rejected with a p-value threshold equal or lower than 0.05. The number of negative samples we use in our experiments is 500.

S5. Computational Resources

Below shows the details of computational resources used in the training of GPO-VAE across the dataset.

Dataset	GPU name	GPU count	GPU memory (MiB)	training runtime	epoch	batch size
rpe1	GeForce RTX 3090	1	24576	1h 25.7m	160	512
K562	GeForce RTX 3090	1	24576	2h 41.4m	166.8	512
Adamson	GeForce RTX 3090	1	24576	47.3m	179	512

S5.1 Post-perturbation Response Prediction Baselines

Model / Method	Runtime		
	K562	RPE1	Adamson
Conditional VAE	2250.4	6250.2	4310.4
SVAE+	2642.4	4169.8	4282.6
SAMS-VAE	2750.2	6848	5040.4
CPA-VAE	3025.6	6667	4832.2
CRADLE-VAE	5446.6	3472.2	6790.2
**GPO-VAE**	**5142.0**	**9684.0**	**2838.0**

S5.2 GRN Inference Baselines

Type	Model / Method	Runtime		
		K562	RPE1	Adamson
random	random100	-	-	0.01
	random1000	0.03	0.03	0.02
	random10000	0.06	0.07	-
observational	PC	10404.01	28897.42	13159.37
	GES	28176.48	12315.27	973.45
	Sortnregress	685.32	132.07	1.59
	GRNBoost	597.11	180.75	24.46
	Notears-lin	22215.33	7528.63	206.38
	Notears-lin-sparse	17001.84	4631.18	212.07
interventional	DCDI-DSF	7298.89	2366.64	1591.84
	DCDI-G	21174.31	8622.35	4847.02
	GIES	4353.01	4775.88	2.31
	DCDFG-MLP	3837.51	2233.34	822.37
	**GPO-VAE**	**5142.00**	**9684.00**	**2838.00**

S6. Detailed Model Architecture

Below shows the detailed model configuration used in GPO-VAE across the dataset.

	guide_kwargs					model_kwargs		
**Dataset**	**basal_encoder_n_layers**	**basal_encoder_n_hidden**	**embedding_encoder_n_hidden**	**embedding_encoder_n_layers**	**n_latent**	**decoder_n_layers**	**decoder_n_hidden**	**n_latent**
rpe1	1	200	400	4	200	1	400	200
K562	1	200	400	4	100	1	400	100
Adamson	1	400	400	4	100	1	400	100

S7. Additional Results

S7.1 Misalignment between biological evaluation and statistical evaluation results

The results below demonstrate a misalignment of biological evaluation and statistical evaluation of biological networks. GT represents the ground truth wasserstein distance-based network by applying a threshold of 0.10, 0.18, and 0.25, respectively. Biological networks included in the evaluation are as follows:

- CORUM :https://mips.helmholtz-muenchen.de/corum/\
- STRING : https://version11.string-db.org/
- RegNetwork : https://regnetworkweb.org/
- BioGRID : https://thebiogrid.org/
- ‘Pool’ represents the union of all biological networks stated above

**Network**	**Wasserstein Distance**	**# Edges**	**pooled biological evaluation**		**CORUM**		**STRING -network**		**RegNetwork**		**Biogrid**	
			precision	recall	precision	recall	precision	recall	precision	recall	precision	recall
GT(>0.10)	0.170	59293	0.289	0.177	0.031	0.216	0.281	0.179	0.002	0.309	0.041	0.193
GT(>0.18)	0.270	16908	0.301	0.053	0.037	0.074	0.293	0.053	0.002	0.088	0.046	0.062
GT(>0.25)	0.358	6956	0.294	0.021	0.040	0.033	0.287	0.021	0.002	0.034	0.044	0.024
pool	0.065	96454	1.000	1.000	0.088	1.000	0.963	1.000	0.002	0.500	0.130	1.000
CORUM	0.073	8528	1.000	0.088	1.000	1.000	0.940	0.086	0.013	0.239	0.458	0.312
STRING	0.066	92886	1.000	0.963	0.086	0.940	1.000	1.000	0.002	0.462	0.101	0.753
Regnetwork	0.085	476	0.500	0.002	0.239	0.013	0.462	0.002	1.000	1.000	0.349	0.013
Biogrid	0.068	12503	1.000	0.130	0.312	0.458	0.753	0.101	0.013	0.349	1.000	1.000

S7.2 GRN topology analysis

The figure below shows the GRN topology analysis of GPO loss objectives in terms of edge weight (causal probability) where darker orange indicates higher probability and lighter orange indicates lower probability:

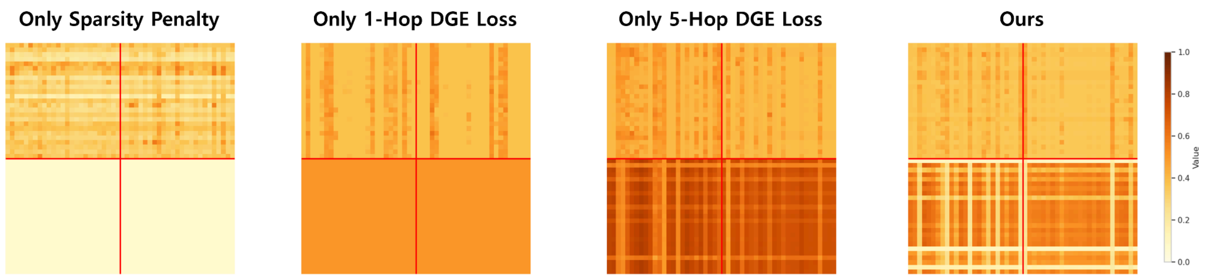

Supplement: btaf256_Supplementary_Data [file btaf256_supplementary_data.pdf]
